# Biology of Anemia: A Public Health Perspective

**DOI:** 10.1016/j.tjnut.2023.07.018

**Published:** 2023-09-29

**Authors:** Gary M. Brittenham, Gemma Moir-Meyer, Kelvin Mokaya Abuga, Ananya Datta-Mitra, Carla Cerami, Ralph Green, Sant-Rayn Pasricha, Sarah H. Atkinson

**Affiliations:** 1 Department of Pediatrics, College of Physicians and Surgeons, Columbia University, New York, NY, United States; 2 Population Health and Immunity Division, Walter and Eliza Hall Institute of Medical Research, Parkville, VIC, Australia; 3 Department of Medical Biology, The University of Melbourne, Parkville, VIC, Australia; 4 Kenya Medical Research Institute (KEMRI)—Wellcome Trust Research Programme, Kilifi, Kenya; 5 Department of Pathology and Laboratory Medicine, University of California, Davis, CA, United States; 6 The Medical Research Council Unit, The Gambia, London School of Hygiene and Tropical Medicine, London, UK; 7 Diagnostic Haematology, The Royal Melbourne Hospital; and Clinical Haematology at the Peter MacCallum Cancer Centre and The Royal Melbourne Hospital, Parkville, VIC Australia; 8 Department of Paediatrics, University of Oxford, Oxford, UK

**Keywords:** anemia, micronutrients, iron, iron deficiency, absolute iron deficiency, functional iron deficiency, infection, inflammation, thalassemia, hemoglobinopathies

## Abstract

Our goal is to present recent progress in understanding the biological mechanisms underlying anemia from a public health perspective. We describe important advances in understanding common causes of anemia and their interactions, including iron deficiency (ID), lack of other micronutrients, infection, inflammation, and genetic conditions. ID develops if the iron circulating in the blood cannot provide the amounts required for red blood cell production and tissue needs. ID anemia develops as iron-limited red blood cell production fails to maintain the hemoglobin concentration above the threshold used to define anemia. Globally, absolute ID (absent or reduced body iron stores that do not meet the need for iron of an individual but may respond to iron supplementation) contributes to only a limited proportion of anemia. Functional ID (adequate or increased iron stores that cannot meet the need for iron because of the effects of infection or inflammation and does not respond to iron supplementation) is frequently responsible for anemia in low- and middle-income countries. Absolute and functional ID may coexist. We highlight continued improvement in understanding the roles of infections and inflammation in causing a large proportion of anemia. Deficiencies of nutrients other than iron are less common but important in some settings. The importance of genetic conditions as causes of anemia depends upon the specific inherited red blood cell abnormalities and their prevalence in the settings examined. From a public health perspective, each setting has a distinctive composition of components underlying the common causes of anemia. We emphasize the coincidence between regions with a high prevalence of anemia attributed to ID (both absolute and functional), those with endemic infections, and those with widespread genetic conditions affecting red blood cells, especially in sub-Saharan Africa and regions in Asia and Oceania.

## Anemia, Iron Metabolism, Absolute, and Functional ID

Iron deficiency (ID), both absolute and functional, is the predominant cause of anemia and poses a significant threat to health and quality of life outcomes [1–3]. ID affects more than 1.2 billion people globally [4], is the leading cause of years lived with disability in low- and -middle-income countries, and is among the top 5 causes of years lived with disability [5].

Reduced iron availability for erythropoiesis can occur because of absolute ID (insufficient iron stores) or functional ID (inadequate iron mobilization despite adequate iron stores) or both together. We emphasize the distinction between these 2 forms of ID in the following sections. Recognizing this difference is critical both for developing more precise approaches to public health interventions for anemia and for clinical care.

## Physiology of Iron Metabolism

**Figure 1:**
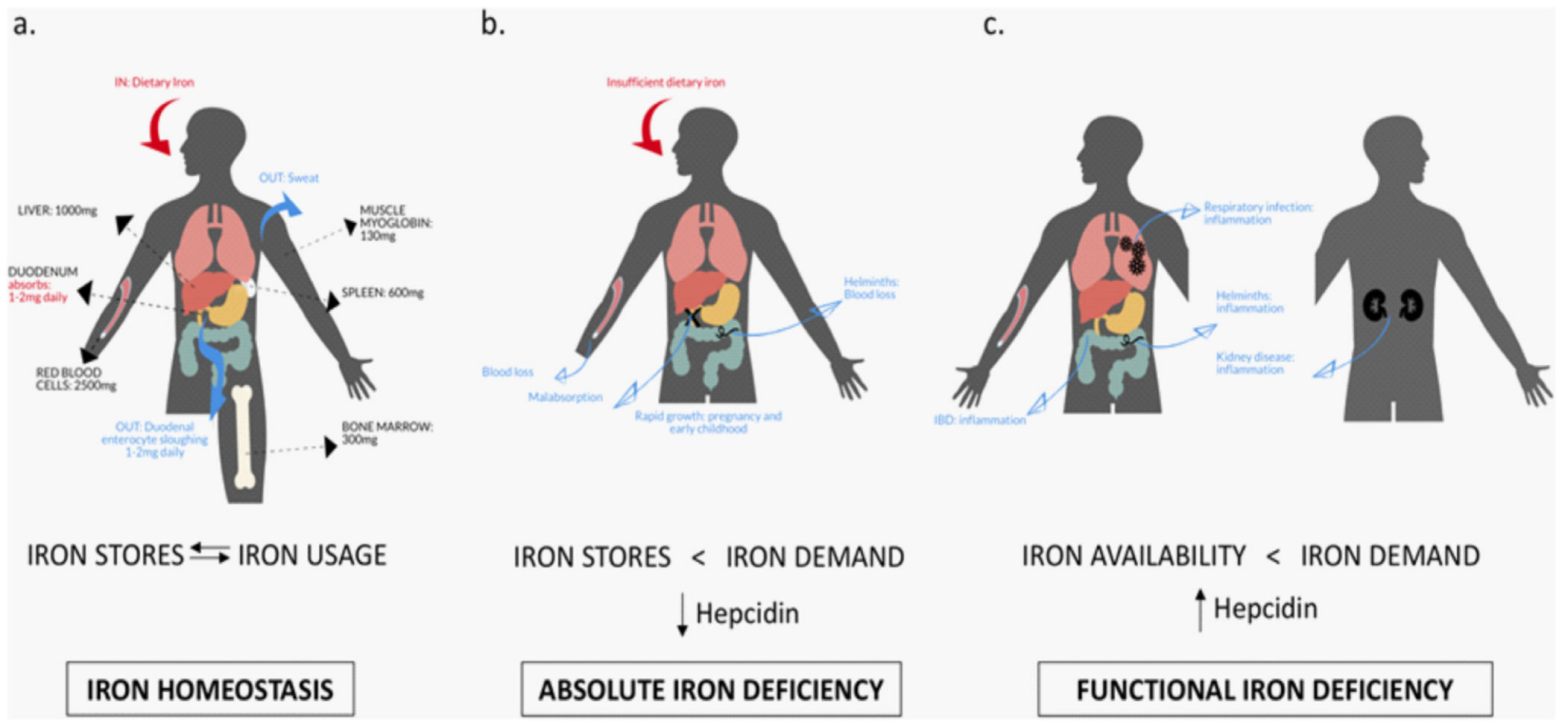
Iron in the body: a balance between iron stores, usage, and hepcidin levels. Panel (A) gives an overview of the flow of iron into the body, where it is contained in the body, and its loss. Absolute iron deficiency (B) can arise when iron stores are insufficient to meet iron demand (hepcidin is downregulated). Functional iron deficiency (C) can develop when iron stores are replete but hepcidin is upregulated due to inflammation, compromising iron supply. Absolute and functional iron deficiency may coexist.

Iron is critical to a variety of core biological functions including energy metabolism, nucleotide synthesis, neurogenesis, and oxygen carriage or storage. Because the only physiological source is dietary intake, iron is an essential nutrient. The demand for iron is the highest from the erythroid compartment for incorporation into hemoglobin (Hb) molecules in red blood cells (RBCs) (Figure 1). Iron is released from RBCs as they senesce, then scavenged by macrophages and promptly made available for distribution to the plasma. Reduced iron availability for erythropoiesis can occur as a result of absolute ID (insufficient iron stores) or functional ID (inadequate iron mobilization despite adequate iron stores).

Absolute ID is especially prevalent in children under 5 y of age and in women of childbearing age, particularly during pregnancy. Functional ID is prevalent in populations in areas where infection is common, in complicated medical and surgical disorders, and in patients receiving erythropoiesis stimulating agents [1]. When iron availability for erythropoiesis is limited, whether from absolute or functional ID, reduced Hb production can lead to poor oxygen transport, and eventually, anemia [6].

Absolute ID can arise from both increased physiological requirements and pathological losses, from insufficient dietary iron intake; insufficient iron absorption; or iron demand exceeding iron supply as a result of growth, pregnancy, hemolysis, bleeding, or treatment with erythropoiesis stimulating agents [1].

Systemic iron regulation converges on a liver-derived regulatory hormone, hepcidin, encoded by the gene hepcidin antimicrobial peptide (*HAMP*) [7]. Hepcidin prevents iron entering the circulation by occluding the cellular iron export channel [ferroportin (FPN)] on the surface of duodenal enterocytes and macrophages while simultaneously initiating FPN ubiquitination and degradation [7]. Liver iron-sensing mechanisms respond to increased iron stores or circulating iron to induce *HAMP* expression, whereas ID, erythropoiesis, and hypoxia downregulate expression of *HAMP* [8]. Systemic inflammation can also induce hepcidin, despite the presence of iron deficits or absolute ID. Even in the presence of replete-iron stores, inflammatory upregulation of hepcidin can limit iron supply and result in functional ID and anemia [1].

## Absolute ID: Iron Requirements and Deficits

Body iron deficits can arise if dietary intake does not meet physiologic requirements (for example, for growth and during pregnancy), or is insufficient to replace losses through shedding of duodenal epithelial cells, sweat, and blood loss [6]. Iron demand is the highest in pregnant women who have a recommended daily intake of 27 mg, followed by premenopausal women (18 mg), infants (11 mg), and is lowest in men (8 mg) [9, 10]. Public health advisory panels advise introduction of iron-rich complementary foods [11] after the period of exclusive breastfeeding. Breast milk provides only 2% of iron required for infants during the critical developmental period between 6 and 24 mo [12].

Heme iron contained in meat is the most efficiently absorbed source of dietary iron (12%–25% bioavailable, whereas nonheme iron may be <5% bioavailable) and is less susceptible to absorption inhibitors such as phytates and tannins [13]. As such, vegetarians who rely on nonheme iron sources have lower iron stores as demonstrated by reduced serum ferritin levels: 29.7 μg/L less than omnivores (95% CI [–39.7, –19.7]) [14]. We discuss the implications of these aspects of iron metabolism for iron bioavailability, and the value of various approaches to improving iron status via food-based interventions or supplementation, in greater detail in the paper on interventions for anemia in this supplement [15].

## Absolute ID: Increased Iron Requirements during Pregnancy and Fetal Development

During pregnancy, there is a more than 9-fold increase in iron absorption to supply a 30% expansion of the Hb mass [16]. Iron lost during bleeding in childbirth, lactation, and erythroid expansion is offset by increased iron absorption and amenorrhea. However, the net pregnancy iron requirement is still substantial (500 mg–1 g) [16,17] and may exceed the amount available from dietary iron absorption. Absolute ID has been considered to account for as much as half of all anemias in pregnancy in low- and middle-income countries [18]. Nevertheless, a recent multi-center, parallel-group, randomized controlled trial in Malawi found that the treatment of anemia in pregnant women with a single dose of intravenous ferric carboxymaltose (20 mg/kg, up to 1000 mg) was superior compared with the standard-of-care oral iron in reducing ID and ID anemia at delivery [19]. Despite the reduction in ID anemia, intravenous iron was not superior to oral iron in reducing the overall prevalence of anemia at delivery, highlighting the complex determinants of anemia in this setting.

In addition to fetal organ development, iron is involved in neuron myelination and neurotransmitter synthesis [20] and affects attention and motor control [21]. Regions such as the hippocampus are particularly iron-dependent [3,22] and deficits in early development produce measurable differences in brain conductivity patterns [22–24]. Importantly, a recent rigorous double-blind, double-dummy, individually randomized, placebo-controlled trial in infants in Bangladesh comparing 3 mo of daily supplementation with iron syrup or multiple micro-nutrient powders with placebo demonstrated that, despite reduced rates of anemia, there was no improvement in cognitive development or neurophysiological outcomes as assessed by event-related potentials [25,26]. This trial provides compelling evidence that any functional deficits caused by ID or anemia cannot be corrected by iron supplementation in infancy [1]. Economic analysis of the same trial using per-disability adjusted life-years averted did not support universal iron supplementation or micronutrient powders as a cost-effective intervention for young children in rural Bangladesh [27]. These findings are also similar to those of an earlier analysis [28], suggesting that universal application of iron supplementation or micronutrient powders to infants may not produce a net benefit in sub-Saharan Africa and may only be cost-effective in regions where adherence is high and there is limited impact of endemic pathogens such as malaria [28].

Table 1 [29–34, 35–63] provides an overview of the risks related to ID in pregnancy and early life. Major factors that contribute to ID are listed, along with their potential effects on health and development and with potential benefits and risks associated with iron supplementation.

## Absolute ID: Iron Losses with Menstruation and Postpartum Hemorrhage

Menstruating women require almost twice as much daily iron as men [7] and are at higher risk of ID [10]. In a study of women in the United Kingdom, the volume of menstrual blood loss explained twice as much variance as differences in the amount of iron in the diet [22]. Absolute ID occurs in two-thirds of women with heavy menstrual bleeding [64,65]. Heavy menstrual bleeding affects up to 20% of women worldwide with a particularly high prevalence in low- and middle-income countries [66–68].

Anemia during pregnancy can increase risk of postpartum hemorrhage (≥500 mL blood loss <24 h after delivery) [18]. Postpartum hemorrhage occurs in 6% of all births globally [69], is a leading cause of maternal death [70,71], and contributes to postpartum anemia in 31%–75% of cases, depending on the volume of blood loss and postpartum care [72].

## Absolute ID: Insufficient Iron Absorption and Bioavailability

After food intake, ingested nonheme ferric iron (Fe3^+^) must be solubilized to ferrous iron (Fe2^+^) in the low-pH environment of the stomach for absorption to occur in the duodenum [73]. The main factors that influence absorption are iron solubility, gastric acidity, duodenal enterocyte surface area, and inflammation [74–76]. Environmental enteric dysfunction, an acquired enteropathy of the small intestine with villus blunting and mucosal inflammation, can compromise nutrient acquisition through loss of absorptive capacity and impaired intestinal function. Environmental enteric dysfunction is widespread among children and adults in low- and middle-income settings [77].

A major contributor to chronic gastritis is *Helicobacter pylori* infection, which occurs in more than half of the global population [78]. *H. pylori* restricts gastric acid secretion and infected individuals have an increased likelihood of developing ID anemia (odds ratio (OR): 1.72, 95% CI [1.23, 2.42]) [79]. In patients receiving proton pump inhibitors or histamine-2 receptor agonists, the reduction in gastric acidity was associated with a higher risk of ID (OR: 2.49, 95% CI [2.35, 2.64]) [76]. Similarly, another study showed that in gastric bypass patients, the incidence of ID increased by 15% at the 2-y follow-up (*n* = 5909) [80].

In the duodenum, villous atrophy occurring in celiac disease is associated with impaired iron uptake due to the reduction in available surface area for nutrient absorption [75,81,82]. Decreased iron absorption often results from simultaneous malabsorption and inflammation (for example, inflammatory cytokines stimulate hepcidin and limit iron mobilization) in Crohn’s disease and ulcerative colitis [83,84].

## Functional ID

Functional ID due to systemic inflammation can develop even in the presence of replete-iron stores. In addition, functional ID can occur in patients with mucosal dysfunction disorders, with complex and chronic medical or surgical conditions, or in patients who require treatment with erythropoiesis stimulating agents [85,86].

Infection prevalence in low- and middle-income countries is a principal cause of systemic inflammation, resulting in functional ID by limiting the absorption and mobilization of iron, thereby also contributing to absolute ID. The effects of inflammation are particularly important if dietary iron is limited and may exacerbate the rate of iron depletion [87]. Iron withholding may have evolved as a host defense mechanism by restricting access to this important element for pathogen metabolism [88]. Even low-grade inflammation elevates hepcidin levels and impairs iron absorption [89,90], suggesting that infection control is essential to resolve ID [91]. For example, systematic reviews indicate improved Hb measures in school-aged children after deworming programs [92,93].

Finally, genetic susceptibility to functional ID is associated with variation in the gene transmembrane serine protease 6 (*TMPRSS6*) that produces the transmembrane protease matriptase-2 [94]. Matriptase-2 negatively regulates hepcidin and mutations in *TMPRSS6* are a rare cause of iron-refractory anemia by elevating hepcidin and preventing iron from moving into the serum [94–97].

## Micronutrients other than Iron Linked to Anemia

The hematopoietic and associated biological systems represent complex networks of intersecting nutrient-dependent pathways and metabolic machinery. As such, deficiencies of multiple micronutrients in addition to iron may influence them. The following section describes our current understanding of the nature and impact of these interactions.

Micronutrients other than iron are essential for the function of a healthy blood system, and thus, adequacy of these nutrients also plays a role in the prevention of anemia. RBC production (erythropoiesis) is a key component for maintenance of an adequate red cell mass in the face of normal patterns of RBC loss through either removal of aged or damaged cells from circulation or to offset blood loss. Because the lifespan of normal red cells is about 4 mo in health, erythroid precursors in the bone marrow require sufficient amounts of key nutrients to undergo rates of proliferation that enable the replacement of ~1% of the total red cell mass daily.

The rates of mitosis of nucleated red cell precursors require adequate production of nucleic acids for DNA synthesis. The production of nucleic acids for use during the S-phase of the cell cycle is dependent on the transfer of one-carbon units. This is accomplished by several substituents of folate which, in turn, requires vitamin B12 (cobalamin) as a cofactor [98]. Thus, a sufficient supply of both folate and vitamin B12 are essential for the maintenance of normal rates of erythropoiesis. Consequently, both folate and vitamin B12 deficiencies are leading causes of nutritional anemia caused by the disruption of DNA synthesis (megaloblastic anemia) [99]. Deficiencies of these key nutrients can arise through inadequacy of intake, malabsorption, or increased utilization or demand.

## Biology of Folate and Vitamin B12 Deficiencies

Vitamin B12 and folate deficiencies cause megaloblastic anemias characterized by ineffective DNA synthesis, resulting in inadequate hematopoietic output in the bone marrow. This in turn leads to cells with larger size, an immature nucleus, and relatively greater amount of cytoplasm. The metabolic interrelationship between folate and B12 accounts for the megaloblastic anemia seen in deficiencies of either vitamin, as B12 is required to restore tetrahydrofolate (THF), which receives a one-carbon unit to generate methylene-THF, crucial for thymidine and DNA synthesis [100].

Although other causes of megaloblastic anemias exist (for example, inherited metabolic disorders, rarely in myelodys-plastic syndrome, or from an acquired defect in DNA synthesis as seen in the settings of chemotherapy) [101], deficiencies in folate and vitamin B12 are the most prevalent causes of megaloblastic anemia worldwide [100]. In North America, and many other countries, due to food fortification with folic acid (a precursor of folate), the prevalence of folate deficiency is low. Classically, megaloblastic anemia, as morphologically diagnosed in the bone marrow and peripheral blood smear, occurs due to an asynchronous nuclear and cytoplasmic development resulting in “megaloblasts,” cells with larger size, greater amount of cytoplasm, and an immature nucleus. The relationship between folate and B12 accounts for the megaloblastic anemia seen in both the vitamin deficiencies because B12 is required to restore THF, which ultimately generates methylene-THF, crucial for thymidine and hence DNA synthesis [102]. Anemia develops gradually and patients present with weakness, palpitation, fatigue, and syncope along with jaundice. There is an associated reduction of white blood cell counts and platelet count. With B12 deficiency, another important symptom is neurological disturbances such as numbness, paresthesia, and decreased position sense. Another interesting sign in these patients is yellowish hyperpigmentation of skin. The main laboratory parameter alterations that occur are anemia with increased red cell indices [mean corpuscular volume (MCV) and mean corpuscular hemoglobin (MCH)], changes in red cell size (anisocytosis), and shape (poikilocytosis) along with low serum/plasma levels of B12 and/or folate and low red cell folate. Patients with borderline B12 levels can be followed further with methylmalonic acid (MMA) levels and rising levels of MMA are highly sensitive and specific for B12 deficiency. Plasma homocysteine levels also rise in B12 deficiency, but it is not a specific indicator of B12 deficiency, as it can also be seen in folate deficiency [102].

Table 2 highlights some of the causes of vitamin B12 and folate deficiencies.

## Other Nutrients Linked to Anemia

Deficiencies of other nutrients, although not as common or as critical as folate, vitamin B12, and iron, may at times cause or aggravate anemia. Leading examples include deficiencies of other B-group vitamins (riboflavin, pyridoxine, and thiamine); ascorbic acid; the fat-soluble vitamins A and E; and other trace elements such as copper, zinc, and selenium, as well as protein-energy malnutrition, which is often complicated with multiple associated micronutrient deficiencies, lack of essential amino acids, or both [102].

Deficiencies of these other micronutrients together constitute only a small fraction of the total burden of anemia accounted for by folate, vitamin B12, and iron deficiencies. Although anemia that results from deficiencies of vitamin B12, folic acid, or iron is generally clearly defined and relatively common, the characteristics of anemia that arises from deficiencies of other micronutrients are poorly defined and relatively rare in humans. Moreover, when present, they often exist not as isolated deficiencies of 1 vitamin or 1 mineral, but as a combination of deficiencies resulting from malnutrition or malabsorption. A case in point is protein-energy malnutrition. Although primarily caused by the insufficiency of a macronutrient, the condition is often associated with deficiency of 1 or more essential micronutrients. This contributes to the challenges of determining which abnormalities are a result of a particular deficiency, and further reinforces the need for fully integrating comprehensive nutritional assessment into anemia public health surveillance and clinical care [103], as discussed elsewhere in this supplement. An overview of the less common micronutrient deficiencies associated with anemia follows and is also presented in Table 3.

## Other Water-Soluble Vitamins and Anemia

A range of water-soluble vitamins other than folate and vitamin B12 have been implicated in anemia. The following is a summary of evidence linking other essential nutrients to anemia: Vitamin B6 (pyridoxine): deficiency of vitamin B6 can cause a microcytic anemia with low MCV and MCH concentration. Vitamin B6 deficiency in infants is associated with a hypochromic microcytic anemia. Vitamin B6 is involved in the synthesis of delta-aminolaevulinic acid, the first step in the heme synthesis pathway. Patients who receive some antituberculosis medicines are more prone to develop vitamin B6 deficiencies without current supplementation with vitamin B6 [104]. Chronic goat milk consumption, which is typically associated with folate deficiency, has also been reported to cause pyridoxine deficiency [105]. These patients typically respond to large doses of vitamin B6 along with cessation of goat milk consumption.

Vitamin B2 (riboflavin): riboflavin deficiency can lead to anemia by causing pure red cell aplasia or by inhibiting release of iron from ferritin. These patients typically respond well to riboflavin supplementation. Women with riboflavin deficiency are twice as likely to present with anemia as those with normal riboflavin status [106].

Vitamin B1 (thiamine): a rare cause of megaloblastic anemia is thiamine deficiency. Thiamine deficiency forms part of a childhood syndrome associated with diabetes and sensorineural deafness, typically reported in individuals of Middle- and Far-Eastern origin, and is caused by a defective thiamine transporter, leading to a critical reduction in the generation of ribose, decreased nucleic acid synthesis, and consequent cell-cycle arrest and apoptosis in the bone marrow. Patients generally are dependent on lifelong thiamine administration [107].

Vitamin C (ascorbic acid): although a high percentage of patients with severe vitamin C deficiency (scurvy) are anemic, attempts to induce anemia in human volunteers by severely restricting dietary vitamin C have been unsuccessful. Individuals with severe vitamin C deficiency can develop anemia. The anemia may result from impaired folate reduction leading to deficiency of tetrahydrofolate and causing megaloblastic anemia [108] or can be the result of bleeding arising from scurvy and causing anemia. Patients with folate deficiency associated with vitamin C deficiency only respond to folate supplementation if it is co-administered with vitamin C. Current evidence also suggests a role for vitamin C in iron uptake through transferrin so that concomitant ID can occur in patients with vitamin C deficiency. Patients with genetic defects of the *TMPRSS6* gene often present with iron-refractory ID anemia; these patients may respond to simultaneous administration of iron and vitamin C [109].

## Fat-Soluble Vitamins and Anemia

Chronic deprivation of vitamin A results in anemia similar to that observed in ID. Unlike absolute ID anemia but like functional ID with the anemia of chronic disease, iron stores are increased, and administration of iron alone does not correct the anemia. Vitamin A deficiency may result in impaired iron absorption or utilization [110]. The simultaneous administration of vitamin A with iron typically shows a better response to the treatment of the anemia [111].

Preterm infants may develop hemolytic anemia, which responds to vitamin E supplementation. Among children with sickle cell anemia, those with vitamin E deficiency have significantly more irreversibly sickled cells [112]. Vitamin E deficiency can lead to hemolytic anemia when low birth weight infants consume diets rich in polyunsaturated fatty acids and iron. Patients generally have decreased red cell life span and edema of the hands and feet. After vitamin E therapy, red cell life span typically improves [113,114].

## Other Trace Minerals and Anemia

Copper deficiency has been described in children who are malnourished and, in both infants and adults receiving parenteral alimentation [115,116]. The bone marrow in copper deficiency shows features of myelodysplastic syndrome with ring side-roblasts, cytoplasmic vacuolation, and a decrease in erythroid and granulocytic precursors. Copper deficiency can occur due to the consumption of large quantities of zinc due to the interference with copper absorption and these patients typically present with a microcytic anemia. Administering copper for a 4–12-wk period can fully reverse the anemia and neutropenia [117].

In adult patients with sickle cell disease, selenium deficiency can be a determinant of hemolysis [118].

## Protein Undernutrition and Anemia

In infants and children with protein-calorie malnutrition (for example, kwashiorkor), the Hb concentration may fall to 8 g/dL but some children with kwashiorkor have normal Hb levels, probably because of a decreased plasma volume. The type of anemia is variable, as is its course. The anemia is usually normocytic and normochromic [102]. Once high-protein diets improve nutrition, reticulocytosis develops, followed by a rise in Hb concentration, hematocrit, and RBC count. Still, improvement is slow and during the third or fourth week, another episode of erythroid marrow aplasia may develop when the children are clinically improved, and serum protein levels are approaching normal. An abrupt fall in Hb following protein feeding may be an ominous harbinger of an adverse and even fatal outcome. During repletion, an increase in plasma volume may occur before an increase in red cell volume, and the anemia may seem to become more severe despite reticulocytosis.

The anemia seen in children with protein-energy malnutrition is often multifactorial and its type is therefore variable. Anemia associated with varying severity of protein-energy malnutrition is often associated with coexistent ID. The most common type of anemia is microcytic followed by megaloblastic [119,120].

## Anemia and Infection

**Figure 2:**
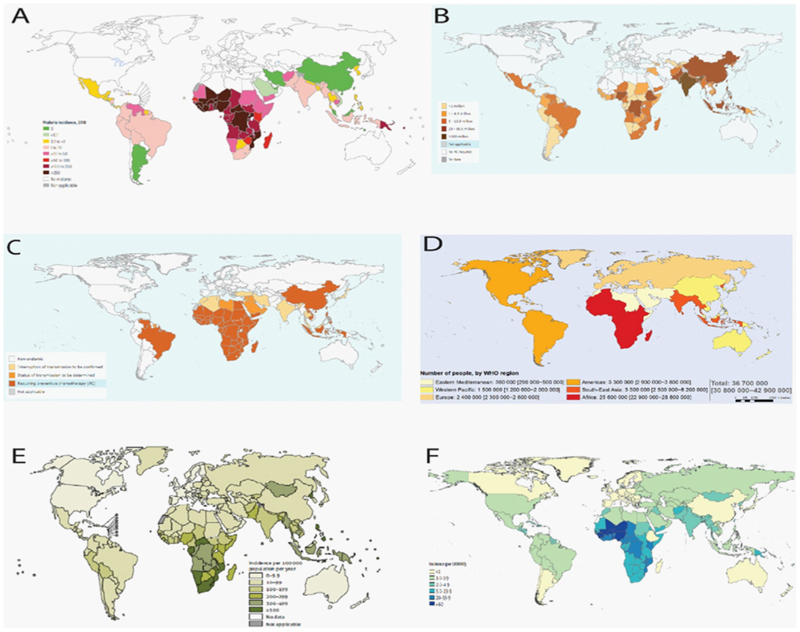
Maps of global prevalence of infections. Panels from L-R, top-bottom: (A) malaria [122] (B) soil-transmitted helminths [129], (C) schistosomiasis [130], (D) HIV [131] (E) tuberculosis [125], and (F) nontyphoidal Salmonella [126].

Infections are responsible for a large proportion of anemia, especially in regions with high anemia prevalence such as sub-Saharan Africa and Oceania. In these regions, young children have the highest prevalence of anemia and total years lived with disability due to anemia [121]. There are several infections that are strongly associated with anemia, including parasitic infections such as malaria, hookworm, and Salmonellae [5,122–126]. Anemia may also complicate severe acute respiratory syndrome coronavirus 2 [127]. Anemia and infections that cause anemia are also major causes of hospitalization and death in children in endemic areas [5,128]. Infections that cause anemia are often localized to specific geographical regions and age groups (Figure 2 and Table 4) [129–131].

## Interactions between Anemia and Infection

The interactions between anemia and infection are complex and may be bidirectional. For example, anemia may influence susceptibility to infection by modulating the host immune response and/or limiting the supply of iron, a growth-limiting nutrient to invading pathogens [132,133]. ID anemia contributes to a suppressed immune response because iron is essential for the development and function of immune cells [133,134] and hemolytic anemia may also modulate immune responses to bacterial infections through heme and heme-oxygenase-mediated dysfunction of phagocytic cells [135,136]. Anemia is also associated with increased gut permeability and may mediate infection with enteric pathogens [137,138]. ID anemia has also been associated with protection against malaria [139,140]; and anemia has been shown to reduce the invasion and growth of malaria parasites in ex vivo studies [141,142], an effect that is reversed by iron supplementation.

## Anemia Induced by Infection

Infections may induce anemia through various mechanisms including inflammation, hemolysis, blood loss, impaired absorption of nutrients, and dyserythropoiesis (Table 5) [143–145]. These mechanisms may also overlap with other infectious, genetic, or nutritional causes of anemia in the same individual or population. As emphasized earlier, sustained expression of hepcidin during infection is the main mechanism leading to the anemia of inflammation. Upon pathogenic invasion, proinflammatory cytokines such as interleukin 6 are induced and mediate expression of acute phase proteins such as hepcidin. As described earlier, hepcidin binds its ligand FPN, the sole iron exporter, resulting in decreased intestinal iron absorption, iron sequestration in macrophages, and reduced erythropoiesis [146]. Although this “hypoferremia of infection” is efficient in limiting iron availability for pathogens, it inevitably over time limits iron availability for RBC production, leading to anemia. Other mechanisms by which infections cause anemia include increased hemolysis of infected or damaged RBCs in schistosomiasis and *Plasmodium* infections [147,148]; blood loss during infections with hookworms and schistosomiasis [147, 149]; impaired absorption of vitamin B12 in *H. pylori* infections [150]; interactions of inflammation and gastrointestinal infections contributing to ID anemia by impairing the absorption of dietary iron causing ID [149,151]; and dyserythropoiesis in *Plasmodium* and some viral infections, including parvovirus [148,151]. Interventions to treat infections may also cause anemia, including antiretroviral therapy such as zidovudine, treatments for hepatitis C such as interferon and ribavirin, and some antibiotics including cephalosporins [152–154].

## Anemia Assessment and Interventions: Influence of Infection and Inflammation

As highlighted elsewhere in this supplement [103], a high prevalence of infection may make the assessment and control of anemia problematic. Complex interactions between the biochemical markers of ID and infection complicate accurate estimation of the prevalence of ID and ID anemia. Acute phase responses to infection, including elevated levels of ferritin, an indicator that when low is commonly used to assess ID, may result in a false assessment of iron sufficiency [155,156]. In malaria-endemic areas, the prevalence of ID may be further underestimated as malaria increases ferritin levels independently of inflammation [157]. The Biomarkers Reflecting Inflammation and Nutritional Determinants of Anemia project utilizes an approach that accounts more fully for the effects of inflammation in the assessment of ID anemia [158]. A detailed description is provided elsewhere in this supplement of the intersection of anemia, infection, and intervention interactions, both clinically and in populations [15]. Notably, interventions such as iron supplementation and fortification may be less effective in areas of high infectious burden where elevated hepcidin due to malaria and/or respiratory infections may limit iron absorption [89,90,159,160]. Oral iron supplementation is also associated with an increased risk of malaria and other infections, and harmful effects on the gut microbiome [161–165]. Malaria control may offer an effective approach to prevention of ID.

Infections may play an important role in the development of more severe anemia. A study among hospitalized children in Malawi showed that malaria and bacterial infection, but not ID, were strongly associated with severe anemia (Hb concentration < 5g/dL) [166]. Another study that included 29,293 children from 16 study sites found that malaria and underweight were most consistently associated with severe anemia (Hb concentration <7g/dL) [167]. Available evidence suggests that the management of malaria and other infections may be important for the prevention of severe anemia, but further research is required in this area.

We outline a number of parasitic, viral, and bacterial infections with known associations with anemia. The importance of each of these infections in contributing toward anemia may vary by age, geographical region, and setting, with young children living in sub-Saharan Africa often carrying the greatest burden. Although discussed individually here, these infectious causes of anemia often coexist in the same populations.

## Parasitic Infections

### Malaria

Malaria is endemic in tropical areas worldwide; for example, across sub-Saharan Africa, an estimated 213 million cases were reported in 2018 [168] with an estimated 24% parasite prevalence in the population [169]. Young children are disproportionately affected.

**Figure 3:**
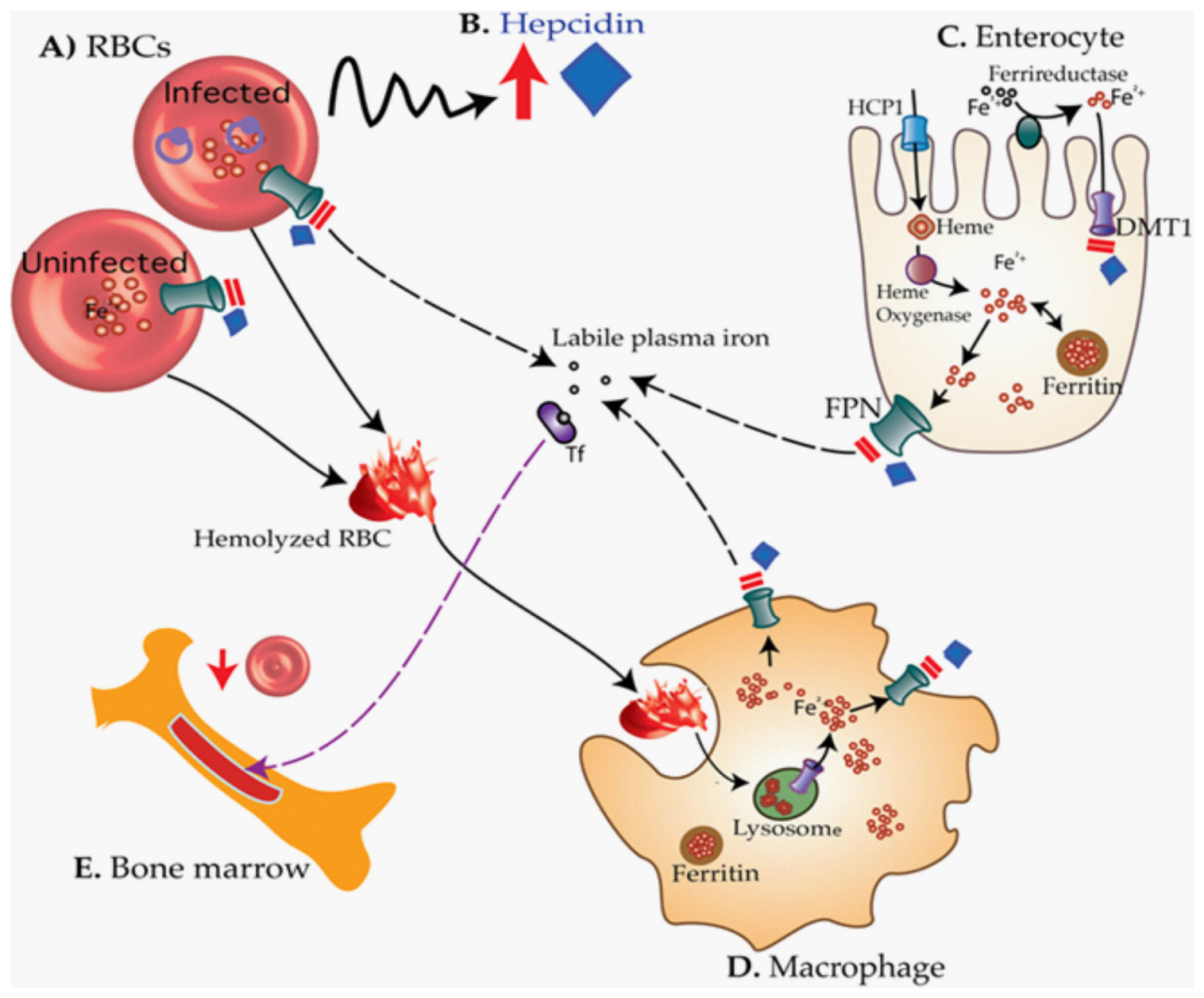
How malaria may cause anemia through iron maldistribution. During malaria infection, blood-stage malaria parasites (A) elicit increased production of hepcidin (B), which in turn block the absorption of iron through ferroportin (FPN) on enterocytes (C). Hepcidin may also degrade ferroportin on both infected and uninfected red blood cells (RBCs), which could lead to accumulation of intracellular iron, oxidative stress, and consequently hemolysis. Hemolyzed RBCs are taken up by the macrophage (D). Hepcidin inhibits recycling of iron recovered from hemolyzed RBCs back into the circulation leading to deficiency of the amount of biologically available iron. Consequently, little iron is available to produce new RBCs by the bone marrow leading to iron deficiency anemia (E) HCP1: heme carrier protein 1. Adapted from Muriuki et al. [172].

Malaria causes anemia through increased intra- and extravas-cular hemolysis of infected and uninfected RBCs, bone marrow dyserythropoiesis, and alterations in the mobilization and utilization of iron [148]. Furthermore, malaria upregulates the production of hepcidin [170,171], inhibiting dietary iron absorption, as well as altering recycling of iron from hemolyzed RBCs [90, 160] resulting in functional ID, reduced erythropoiesis, and anemia of inflammation (Figure 3) [172]. Data suggest that hepcidin may be more highly induced during malaria compared with other infections and that afebrile malaria, prevalent in approximately a quarter of children in sub-Saharan Africa, also raises hepcidin above a threshold associated with impaired iron absorption [90, 160]. Indeed, intermittent preventative treatment of malaria and other control strategies including insecticide-treated nets, and insecticide residual spraying are associated with reduced functional ID and anemia [173–175]. An intervention that halves risk of malaria episodes could reduce the prevalence of ID in children in Africa by as much as 49% [176].

### Other Parasitic Infections

Hookworms (*Ancylostoma duodenale* and *Necator americanus*) and schistosomiasis are important causes of anemia that often coexist in the same regions as malaria [177,178]. Estimates suggest that hookworms afflict ~472 million people contributing to 4 million disability-adjusted life years with the highest prevalence in sub-Saharan Africa and parts of Asia [5].

Hookworms cause anemia from chronic blood loss due to the blood feeding of intestinal stages of the parasite. The degree of anemia is proportional to the density of hookworms harbored. Moderate and heavy hookworm infestations are associated with reduced Hb levels and anemia [179]. However, unlike malaria and other infections, hookworm infection does not influence hepcidin concentrations so iron absorption is not impaired [160].

Schistosomiasis (*S. mansoni* and *S. haematobium*) is another common parasitic disease; ~228 million individuals were affected in 2018 with 90% living in Africa [178,180]. Schistosomiasis is associated with reductions in Hb concentrations [181,182] that improve with treatment [183]. In schistosomiasis, anemia may be caused by extracorporeal blood loss due to translocation of eggs across the bladder or intestinal walls, cytokine-mediated dyserythropoiesis, autoimmune hemolysis, or portal hypertension due to hepatic granulomas [147,184].

## Viral Infections

### HIV

Approximately 38.8 million people were living with HIV in 2015 with about 70% living in sub-Saharan Africa [123]. The prevalence of anemia is high among people living with HIV, reaching up to 80%–90% in low- and middle-income countries [185]. HIV-related anemia is strongly associated with a poor prognosis including accelerated disease progression and a 3.5 times (95% CI: 2.48, 4.94) increased risk of all-cause mortality in meta-analyses [186].

HIV infection causes anemia through a wide range of mechanisms, including direct infection of hematopoietic precursor cells, immune-mediated cytopenias and anemia of inflammation, opportunistic infections such as Epstein–Barr virus and cytomegalovirus, and the direct effect of antiretroviral drugs such as zidovudine. Elevated ferritin levels are associated with a two-fold increased risk of mortality in meta-analyses and there are concerns regarding the safety of iron supplementation in individuals living with HIV infection; few studies have investigated this potential interaction [187].

### Hepatitis C virus (HCV)

Globally, an estimated 71 million people have HCV, which is associated with acute and chronic hepatitis and liver cancer [188]. HCV infections can induce autoimmune hemolytic anemia [189], although the precise mechanisms remain unknown. In addition, the treatment of HCV is associated with an increased risk of anemia, and more than 50% of all treated patients may develop anemia [153]. Drugs to treat HCV infection may suppress RBC production (as with interferon) or induce hemolysis (as with ribavirin) [153,190].

### COVID-19

The WHO declared COVID-19 a global pandemic in March 2020 [191]. Accumulating evidence suggests that dysregulation of iron homeostasis is a hallmark of the infection. Patients with COVID-19 with severe disease or poor outcomes have been reported to have reduced Hb, higher plasma ferritin, and lower iron levels [192,193]. Whether low iron and/or Hb levels are consequences or modulators of pathogenesis of COVID-19 disease remains unknown [194].

## Bacterial Infections

### Tuberculosis (TB)

An estimated 10 million new cases of TB and 1.2 million TB-related deaths were reported in 2018 [125]. Anemia is highly prevalent among individuals with TB disease affecting more than 90% of patients in some studies [195]. TB-associated anemia is predominantly due to chronic inflammation characterized by increased levels of C-reactive protein and proinflammatory cytokines [196–198]. Other causes include nutritional deficiencies, such as folate and vitamin B12 deficiencies, autoimmune hemolytic anemia, and bone marrow fibrosis and dysfunction [199]. Antitubercular treatment is associated with resolution of anemia in approximately two-thirds of patients [196,200].

### Other bacterial infections

Invasive bacterial infections account for 6%–15% of hospital admissions with high mortality [201–203]. Epidemiological studies have reported a strong association between invasive bacterial infections and anemia [166,201,204], particularly with severe anemia. The direction of causality and the precise mechanisms underlying this association remain unknown. Bacterial infections may cause anemia through hypoferremia of infection, bacterial hemolysins causing hemolysis, and bone marrow suppression by proinflammatory cytokines [132]. Some antibiotics are also associated with hemolytic anemia [154]. However, anemia may increase risk of bacterial infections through iron dysregulation, increased gut permeability, hemolysis, and immune dysfunction [205].

**Figure 4:**
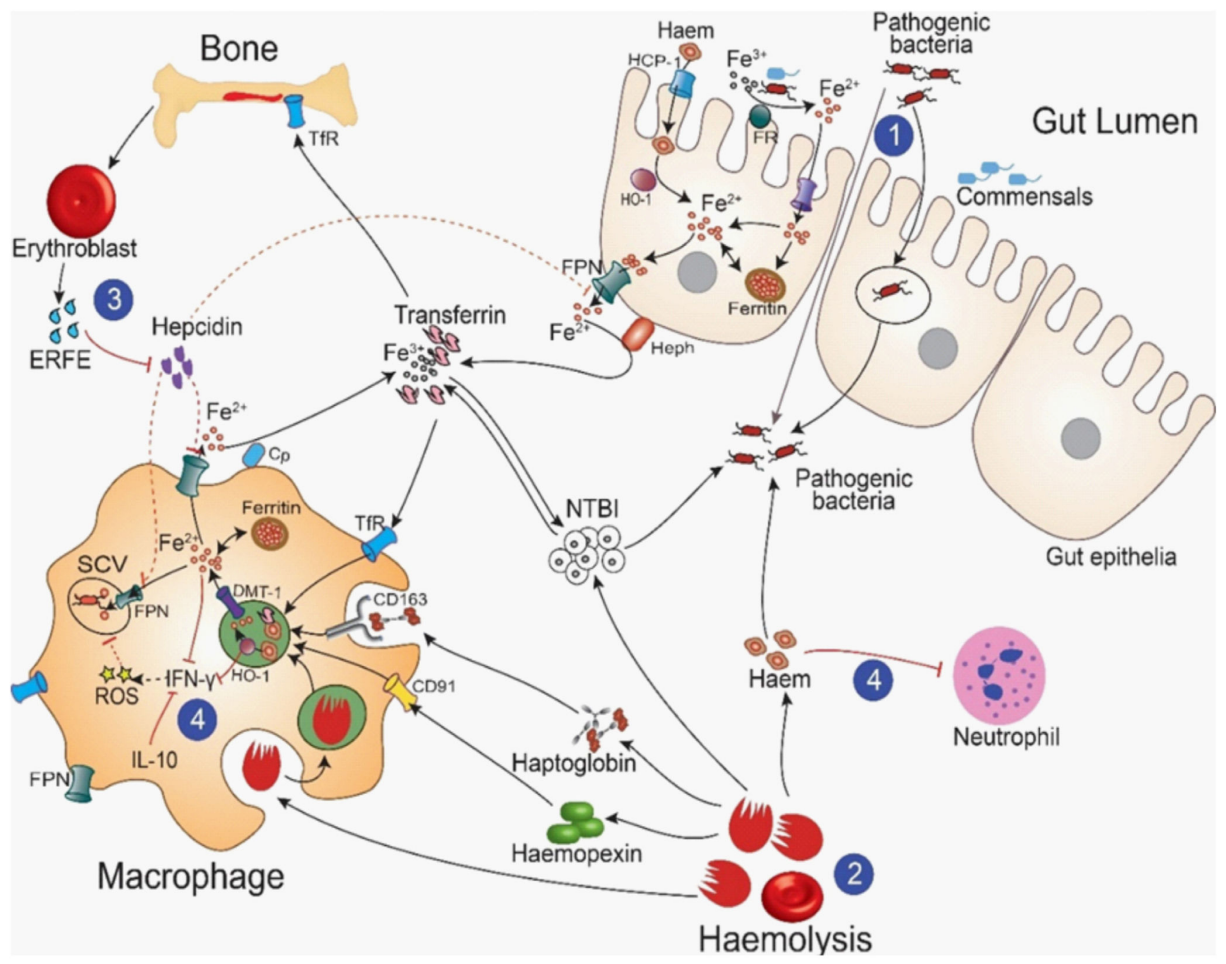
The link between anemia and invasive bacterial infections. Anemia may increase risk of bacterial infections through various mechanisms including *1*) increased gut permeability of enteric pathogens; *2*) increased hemolysis and destruction of red blood cells, which increases availability of NTBI and free heme; *3*) reduced hepcidin levels due to increased production of ERFE, which suppresses hepcidin, increasing iron export from storage cells through ferroportin (FPN), the sole iron exporter; and *4*) immune dysfunction including decreased production of interferon gamma (IFN-γ) and neutrophil mobilization. ERFE, erythroferrone; NTBI, non-transferrin-bound iron; ROS, reactive oxygen species; TfR: transferrin receptor; SCV: Salmonella-containing vacuole. Adapted from Abuga et al. [205].

Iron and heme are important for bacterial growth and are acquired through*1*) chelation of iron or heme using siderophores and hemophores or*2*) direct uptake of iron, Hb, and heme moieties. As an immune response, hepcidin is expressed to limit iron availability to bacteria, exert antimicrobial activity, or both [206–209]. The hypoferremia of infection can be counterproductive during coinfections with intracellular bacteria [210–212], including *Listeria monocytogenes, Mycobacteria tuberculosis*, and nontyphoidal Salmonella (NTS). Low hepcidin levels and increased expression of FPN may control the replication of *M. tuberculosis* and *Salmonella Typhimurium* [213–215], but further work is needed to determine whether FPN transports iron into or out of phagolysosomes [215,216]. Figure 4 shows a summary of the link between anemia and bacterial infections.

### Anemia and the Gut Microbiome

The gut is colonized by a plethora of normal flora and pathogenic bacteria, which play an important role in maintenance of health and disease pathogenesis. High plasticity in the composition of the gut microbiome is present both within and between individuals and populations [217]. The gut microbiome may promote ID in individuals having low iron diets [218]. On the other hand, both ID anemia and iron supplementation are associated with gut dysbiosis and an increased presence of pathogenic microbes. ID impairs gastrointestinal immune protection whereas iron supplementation may enhance the growth of pathogenic bacteria [162,164,165,219,220]. Some bacteria, such as *H. pylori*, are associated with anemia (including pernicious anemia and ID anemia) by impairing absorption of vitamin B12 and other nutrients [150].

### Anemia and Genetic Conditions

**Figure 5:**
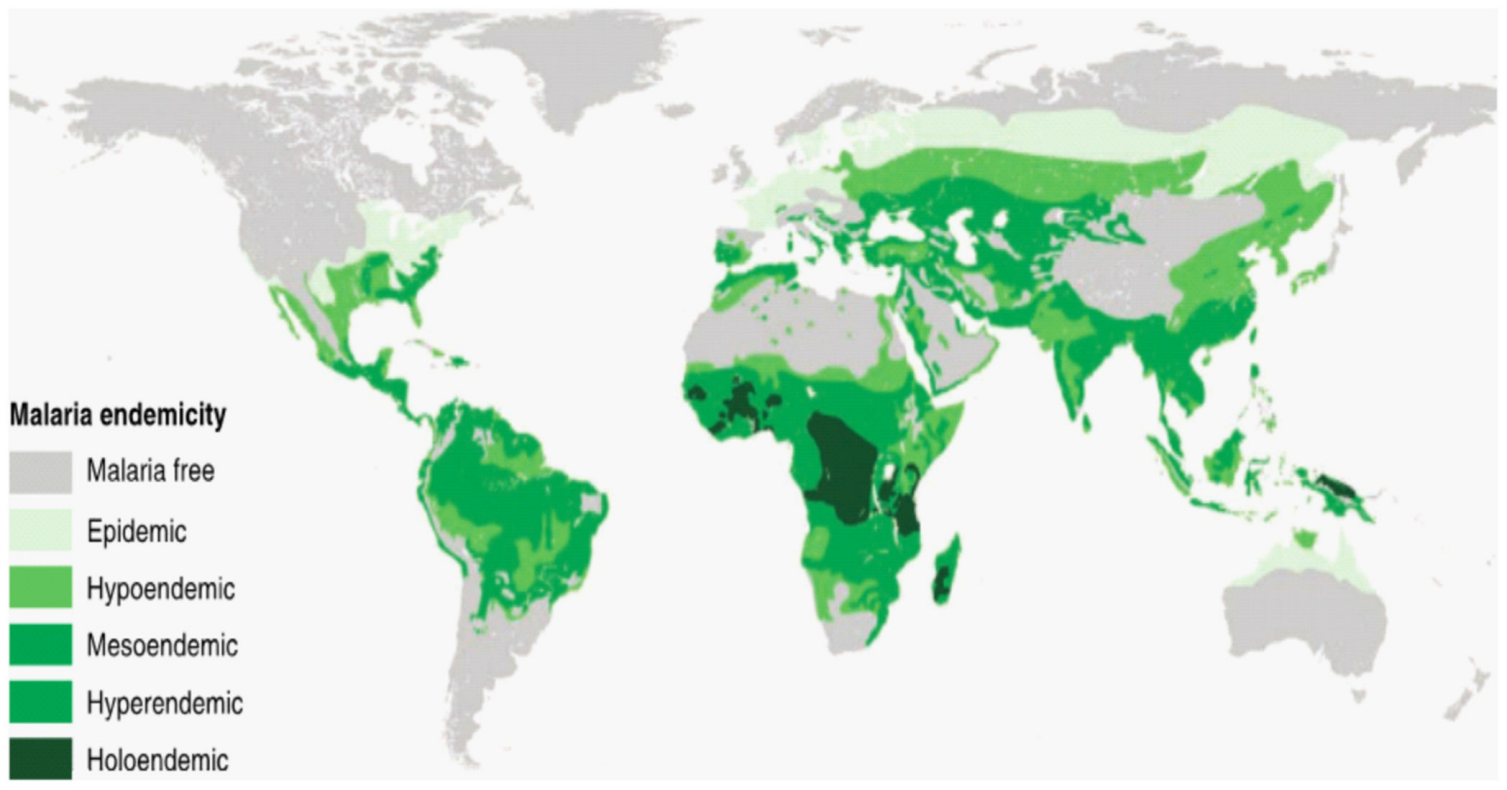
A historical global map of malaria endemicity. This map represents malarial endemicity. Source: Piel et al. [221, page 3]. Malaria endemicity is classified by the parasite rate, that is, the proportion of the population found to carry asexual blood-stage malarial parasites. Holoendemic: a parasite rate <10%, mesoendemic: a parasite rate ≥10% and <50%, hyperendemic: a parasite rate ≥50% and <75%: holoen-demic a parasite rate ≥75%.

Inherited blood disorders that cause anemia at frequencies greater than those from random mutation are predominantly associated with resistance to *Plasmodium falciparum* malaria (Figure 5) [221,222]. All malarial infections involve the RBC, the site of parasite invasion and of proliferation during the blood stage of malarial infection. The inherited RBC conditions associated with malaria include abnormalities of Hb synthesis (α- and β-thalassemia) (Supplemental Figures 1 and 2), Hb structure (Hb S, C, and E; Supplemental Figure 3), red cell enzymes (glucose-6-phosphate dehydrogenase [G6PD] deficiency; Supplemental Figure 4), or the RBC membrane (hereditary spherocytosis, elliptocytosis, and ovalocytosis). None of these genetic disorders prevent *P. falciparum* infection; each offers different mechanisms and degrees of protection against malarial complications (Supplemental Table 1) [222]. The importance of these inherited red cell abnormalities as causes of anemia depends upon the specific genetic condition, the pattern of inheritance and the populations, geographic areas, and settings examined. Each year, an estimated 500,000 children are born with severe anemia from genetic disorders of the RBC, with 80% in low- and middle-income countries in sub-Saharan Africa, the Mediterranean region, the Middle East, South and Southeast Asia, and Oceania [223].

## Genetic Disorders of Hb Synthesis and Structure: Hemoglobinopathies

### Genetic disorders of Hb synthesis (thalassemia)

The hereditary disorders of globin synthesis are β- and α-thalassemia, named for the globin chains affected (Supplemental Figure 5) [224]. Various forms of thalassemia are the most common genetic disorders in the world [225].

#### β-thalassemia

Normal Hb synthesis requires 2 β-globin genes, 1 on each chromosome 11 (Supplemental Figure 5) [224]. In heterozygous β-thalassemia (β-thalassemia trait), the partial or complete loss of globin production from a single β-globin gene does not result in anemia. In homozygous or compound heterozygous β-thalassemia, with both β-globin genes affected, moderate to severe anemia develops (thalassemia intermedia to thalassemia major), depending on the magnitude of the loss of β-globin production and the inheritance of phenotypic modifiers (Supplemental Figure 6) [224–226]. If a stem cell transplant is not feasible, the most severe forms of β-thalassemia major require lifelong transfusion and iron chelation for survival (Supplemental Figure 6) [226]. Patients with thalassemia intermedia have increased erythropoiesis, relative hepcidin suppression, and increased iron absorption that may result in iron overload [227,228].

#### α-thalassemia

Normal Hb synthesis requires 4 α-globin genes, 2 on each chromosome 16 (Supplemental Figure 5) [224]. The types of α-thalassemia resulting from deletions or mutations of 1 or 2 of the 4 α-globin genes cause minimal or mild anemia that is difficult to distinguish from the anemia of ID or of inflammation without genotyping. These forms of α-thalassemia reach a prevalence of 40% or more in Africa and up to near 90% in some areas of southeast Asia. Forms of α-thalassemia resulting from deletions or mutations of 3 of the 4 α-globin genes (Hb H disease) produce severe anemia [224]. Loss of all 4 functional α-globin genes results in still birth of a hydropic infant (Hb Bart’s hydrops fetalis).

### Genetic Disorders of Hb Structure

At the population level, the principal hereditary disorders of globin structure result from single mutations in the β-globin gene, producing Hb S (β6Glu→Val), Hb C (β6Glu→Lys), and Hb E (β26Glu→Lys) [223]. The carrier state (heterozygosity) for Hb S, C, and E does not cause anemia. Homozygous Hb S (Hb SS: sickle cell anemia) results in a severe chronic hemolytic anemia with chronic vaso-occlusive organ damage and recurrent painful episodes (Supplemental Table 2) [229]. Homozygous Hb C produces a moderate hemolytic anemia, typically with splenomegaly [225]. The Hb E β-globin gene is synthesized at a reduced rate, resulting in a phenotype resembling a mild thalassemia. Homozygotes for Hb E resemble β-thalassemia heterozygotes [225].

### Coinherited Genetic Disorders of Hb

Globin genes are inherited independently and combinations of hemoglobinopathies may result (Supplemental Table 2). The anemia of the sickling disorder, Hb S-β-thalassemia may be as severe as that of homozygous sickle cell disease (Hb SS) or milder, depending on the magnitude of the loss of β-globin production, coinheritance of α-thalassemia, and phenotypic modifiers [225]. Hb SC produces a milder anemia and fewer painful episodes than Hb SS [226]. At the population level, Hb E β-thalassemia is one of most prevalent syndromes, with extraordinary phenotypic variability, ranging from mild thalassemia intermedia to severe β-thalassemia major (Supplemental Figure 6) [226].

### Genetic Disorders of RBC Enzymes

Although numerous inherited disorders of RBC enzymes have been identified, G6PD deficiency is the most common metabolic disorder of RBCs and the most important at the population level (Supplemental Figure 4) [230]. Resulting from a variety of mutations of differing severity in the X-linked G6PD gene, hemizygous males, homozygous females, and (as a result of genetic mosaicism from X-chromosome inactivation) some portions of heterozygous females are affected [231]. G6PD-deficient individuals normally are asymptomatic and not anemic, but episodically can develop an acute hemolytic anemia after exposure to fava beans or other food, infections, and certain drugs [230]. In particular, testing for G6PD deficiency is recommended before the administration of primaquine or tafenoquine for treating *P. vivax* hypnozoites [232].

### Genetic Disorders of the RBC Membrane

Inherited abnormalities of the RBC membrane, including hereditary spherocytosis, elliptocytosis, ovalocytosis, and related disorders, resulting from mutations in genes encoding membrane proteins (α-spectrin and β-spectrin, actin, band 3, ankyrin, and others), may cause hemolytic anemia ranging in severity from minimal to severe [233]. Their distribution is worldwide, but these disorders are most commonly found in Africa, Southeast Asia, and Oceania. Among the best characterized is the autosomal dominant Southeast Asian Ovalocytosis, which causes only a minimal to mild anemia in heterozygotes but seems to provide some protection against both *P. falciparum* and *P. vivax* malaria; the homozygous form is apparently almost always lethal [234].

### COVID-19: Interactions with Inherited Blood Disorders

Many patients with inherited blood disorders, including thalassemia, sickle cell disease, and RBC enzyme and membrane disorders, are at increased risk of developing severe complications from COVID-19 [235].

## Summary

### Anemia, iron metabolism, absolute and functional ID

ID can be a result of insufficient iron stores, inadequate iron mobilization, or a combination thereof. When hepcidin is suppressed under conditions of low iron, heightened erythropoiesis, or hypoxia, iron absorbed from the gut and released from iron stores increases, improving the supply for RBC production. Several regulatory pathways converge on the gene *HAMP* and by iron sensing or inflammation upregulate hepcidin production, which blocks FPN from exporting iron, decreasing the supply for RBC production. Understanding the complex etiology of ID and anemia is essential to diagnosis, treatment, and management. As a leading cause of anemia in low- and middle-income countries, management of ID, both absolute and functional, is a public health priority. In each public health setting, programs to ameliorate ID anemia need to recognize that only a minority of all anemia is responsive to dietary measures or iron supplementation because of the overlap between the effects of poor nutrition, chronic infections, inflammation, environment, genetic disorders, and other factors.

### Micronutrients other than iron linked to anemia

Although the evidence and prevalence estimates of the contributions of nutrients other than iron, folate, and vitamin B12 are limited, a convincing case exists for a greater appreciation of multiple nutrients acting singly or via interactions within the hematological network. From a research perspective, we need to further understand the nature of the interactions of multiple micronutrients within relevant systems and the application of the resulting knowledge to improve nutritional assessment and interventions in the context of anemia.

### Anemia and infection

Preventing and treating infection, and especially malaria, should be an integral strategy for the management of ID and anemia in areas of high infectious disease burden. Prevention and treatment of infection are especially important for children living in sub-Saharan Africa, in conjunction with precautions to allay concerns regarding the safety and efficacy of iron interventions [15,160,165,236]. Research priorities about the intersection of anemia and infectious diseases include*1*) quantifying the effects of infection on risk of anemia and*2*) the proportion of anemia that would be prevented by infection control [237].

### Anemia and genetic conditions

Hundreds of hereditary disorders causing anemia have been identified that may require diagnosis, management, and medical resources available only in specialized centers. Progress in understanding the underlying mechanisms of genetic disorders has been made [238] and new therapeutic approaches are entering human trials [229,239–241]. Numerous research priorities need to be recognized for an expanded understanding of inherited blood disorders. These include better genetic epidemiology, micromapping of red cell variants [241]; and the development of improved methods for reliable genotyping [242], diagnosis, genetic counseling, treatment, and training in each country. Investigation is needed into risk of iron overload from iron supplementation in carriers or those more severely affected by the genetic disorders of the RBC. Overall, the genetic disorders of the RBC constitute a neglected global health problem that needs improved prevention and management policies [243].

In summary, progress in understanding the biological mechanisms underlying anemia has steadily increased the awareness of the extensive overlap between the common causes of anemia in settings with a high prevalence of ID (both absolute and functional), those with endemic infections, and those with widespread genetic conditions affecting RBCs. Consequently, in low- and middle-income countries, greater progress in reducing the prevalence of anemia will require public health programs that are not limited to nutrient interventions alone and that assess the role of genetic conditions and incorporate measures to control infection.

## Supplementary Material

Supplementary data to this article can be found online at https://doi.org/10.1016/j.tjnut.2023.07.018.

Supplementary Material

## Figures and Tables

**Table 1:** Overview of risks related to iron deficiency in pregnancy and early life

	Mother: pregnancy	Infant: birth-6 mo	Infant/toddler: 6-24 mo	Mother and infant
A. Risks for ID	Increased iron requirement [16] not met by increased absorption [29,30]. Maternal diabetes [31]. Excessive bleeding during childbirth not treated with transfusion, infection, or inflammation [69]. Soil-transmitted helminth infections. Deworming is associated with 23% reduction in anemia during pregnancy [32].	Cord clamping not delayed [3,33] Insufficient fetal iron stores [3,17] Exclusive breastfeeding in ID-susceptible infants [34] Premature birth, lowbirthweight [21,24] Rapid growth [35]	Low bioavailability of complementary foods [12]. Insufficient fetal iron stores [3,17]. Male infants [35–37].	Mothers who are iron deficient have increased risk of developing antenatal [38] and postpartum depression [39]. Maternal depression is associated with a variety of negative effects on infant outcomes, including cognitive deficits [40,41] and diminished quality of maternal interaction [42-44].
B. Risks from ID	Mortality (in cases where hemoglobin < 70 g/L) [31,45]. Increased fatigue, cognitive problems, and mental health [46,69]. Hemoglobin <110 g/L is associated with increased postpartum hemorrhage, preeclampsia and transfusion [70].	Altered myelination, impaired hippocampal development, impaired dopaminergic neurotransmission [31, 36]. Abnormal auditory neural maturation [47]. Low birthweight, prematurity [48,70]. Intrauterine growth restriction [48].	Reduced maternal facial recognition [23]. Reduced maternal voice recognition [24]. Abnormal neurobehavioral processing [49]. Adverse effects on central nervous system development at 6-12 mo post ID [50].	
C. Iron Supplementation	Increased hemoglobin and decreased anemia [2,51]. IV iron is superior to oral iron to improve ID in pregnant women [52-54].	Maternal iron supplementation: Increases infant hemoglobin concentrations at birth and postpartum [55] Increases infant birthweight and gestational age [55] Increases infant birthweight [51] and decreases small-for-gestational-age infants [56]	Reduced ID, higher hemoglobin, higher ferritin [57]* Lower levels of ID and ID anemia in infants who received supplementation but no difference in cognitive, developmental or growth outcomes relative to placebo [1]. It is also important to note that iron supplementation can modify the intestinal microbiome because the majority of iron from supplements is not absorbed and can instead be used by pathogenic gut bacteria [58-60]. Iron supplementation in clinical trials has been associated with increased rates of diarrhea in some cases [61,163,164] and interventions should be considered in the context of anemia prevalence, anemia severity, infection prevalence and intervention coverage [28]. Iron supplementation in malaria-endemic regions should be coupled with anti-malarial measures [62,63].	

**Table 2:** Causes of vitamin B12 and folate deficiencies

Folate deficiency (less common)	Vitamin B12 deficiency
1. Risk factors for reduced intake:	1. Malabsorption:
a. Elderly, excessive alcohol intake, and poverty	a. Intrinsic factor defect in pernicious anemia (autoimmune gastritis)
b. Premature infants	
c. Synthetic diets, parenteral nutrition, and exclusive goat’s milk intake	b. Hereditary intrinsic factor deficiency
	c. Stomach and small bowel resections
d. Hemodialysis	d. Zollinger-Ellison syndrome
	e. Pancreatic exocrine insufficiency
	f. Blind loop syndrome with bacterial overgrowth
	g. Crohn’s disease
	f. Fish tape worm infestation
	i. Intestinal parasitic infection
2. Increased demand:	2. Reduced intake:
a. Pregnancy	a. Vegetarians, and in particular, strict vegans
b. Growth spurts in puberty	
c. Skin lesions: exfoliative dermatitis	b. Breastfeeding infants of vegan mothers
d. Hemolytic anemias	
3. Malabsorption:	
a. Celiac disease	
b. Tropical sprue	
4. Drug induced:	
a. Anticancer agents (methotrexate, etc.)	
b. Anticonvulsants	
c. Antibiotics (trimethoprim, sulphonamides, etc.)	

**Table 3:** Anemia linked to micronutrients and trace elements [121]

Nutrient	Function	Hematologic consequences	Mechanism of anemia	Sources	Conditions leading to deficiencies	Other consequences of deficiencies
Vitamin A	Antioxidant Growth and differentiation of epithelial cells Development of visual pigments in the retina Maintenance of proper immune function	Microcytic and hypochromic anemia with anisocytosis and poikilocytosis	Impairment of iron absorption and utilization Also plays a role in growth and differentiation of erythroid precursors	Beef liver, salmon, and dairy products, and provitamin A carotenoids in green leafy vegetables, orange and yellow vegetables, carrot, apricots, and mangoes	Fat malabsorption syndromes (celiac disease, pancreatic insufficiency), worm infestation in LMICs	Night blindness, xerophthalmia, skin keratinization
Thiamine (Vitamin B1)	Coenzyme in pyruvate and alpha-ketoglutarate dehydrogenases and transketolases: decarboxylation and group transfer reactions in carbohydrate metabolism pathways	Megaloblastic anemia	Induces cell-cycle arrest or apoptosis in marrow cells	Whole grains, meat, fish, legumes, seeds, nuts	Inborn errors of metabolism, boiling milk	Beriberi, Wernicke-Korsakoff syndrome
Riboflavin (Vitamin B2)	Coenzyme in redox reactions and mitochondrial electron carriage flavin mononucleotide and dinucleotide	Vacuolated red cell precursors and red cell aplasia, normocytic anemia	Impaired iron release from ferritin Induction of glutathione reductase deficiency	Cheese, almonds, beef and lamb, mackerel, eggs, pork, mushrooms, spinach	Alcoholics, dietary deficiency	Cheilosis, glossitis, stomatitis
Niacin (Vitamin B3)	Electron transfer and redox reactions (Nicotinamide adenine dinucleotide/Nicotinamide adenine dinucleotide phosphate)	Anemia	Unknown	Dairy, eggs, enriched breads and cereals, fish, lean meat, nuts, and legumes	Alcoholics, homelessness, malabsorption, children who are malnourished	Pellagra: diarrhea, dementia, dermatitis, death Peripheral neuropathy
Pantothenic acid (Vitamin B5)	Acyl carrier in lipid biosynthesis pathways	Anemia in animal models; not reported in humans when artificially induced	Unknown.	Meats, vegetables, Brewer’s yeast, and grains	Rare	Poor wound healing, lipid metabolism disorders, susceptibility to infections
Pyridoxine (Vitamin B6)	Decarboxylation and transamination reactions in amino acid synthesis	Microcytic hypochromic anemia, ring sideroblasts	Impairment of hemoglobin synthesis via the porphyrin synthetic pathway where pyridoxine acts as a cofactor	Meats, whole grains, nuts, and vegetables	Antitubercular agents (Isoniazid), protein-energy malnutrition, malabsorption, excessive alcohol intake	Cheilosis, glossitis, stomatitis, seborrheic dermatitis, peripheral neuropathy
Vitamin C	Hydroxylation of lysine and proline in collagen synthesis	Megaloblastic anemia	Failure to synthesize tetrahydrofolate or protect it from oxidation ultimately results in megaloblastic anemiCompromised intestinal iron absorption due to failure of reduction of ferric to more soluble ferrous state	Citrus fruits, peppers, guava, kale, broccoli, tomatoes, peas	Exclusive cow’s milk feeding, excessive alcohol intake, elderly on exclusive fast food diet	Scurvy
Vitamin E	Antioxidant Role in cell signaling	Hemolytic anemia, thrombocytosis	Anemia often is associated with fragmentation and other morphologic alterations of the erythrocytes as alpha to copherol is an antioxidant preventing oxidant damage to red cell membrane.	Nuts, green vegetables, sunflower seeds	Chronic pancreatitis, cystic fibrosis, cholestasis, primary biliary cirrhosis, short bowel syndrome, Crohn’s disease	Neurological dysfunction, visual problems, and myopathies
Copper	Iron metabolism, bone growth, immune function	Ring sideroblasts, macrocytic, normocytic, and less commonly microcytic anemia, vacuolated erythoid and granulocytic precursors, neutropenia Hemosiderin laden plasma cells and hypogranular and hypolobated neutrophils	Hampers iron absorption and utilization	Beef liver and heart, red meat	Wilson’s disease, nephrosis, protein losing enteropathies	Neurological and skeletal abnormalities, depigmentation of hair, cardiac defects
Zinc	Wound healing, maintenance of eye and skin health, component of numerous zinc containing metalloproteinases and enzymes	Microcytic anemia due to concomitant iron deficiency	Hinders iron absorption	Meats, grains, nuts, oysters, milk products, bread	Low intake, high cereal fiber and legume diet, mutation in solute carrier family 39 member 4 (SLC39A4), a zinc uptake protein	Growth retardation, acrodermatitis enteropathica, impaired taste sensation, and impaired wound healing
Selenium	Acts as an antioxidant, needed for thyroid hormone synthesis	Macrocytic anemia or hemolytic anemia	Unknown	Liver, pork, fish, eggs, whole grains, nuts	Low intake in areas where soil is selenium deficient	Cardiac and skeletal muscle myopathies

LMIC, low- and middle-income country.

**Table 4:** Estimated global prevalence of selected infections by age in millions

Infection	All ages (95% CI)	Young children ≤5 y (95% CI)	School-age children, 5–14 y (95% CI)	Adolescents and adults ≥14 y (95% CI)	Reference
Malaria	228 (206, 258)	23.8^1^	N/A	N/A	[122]
Hookworm	451 (425, 479)	N/A	N/A	N/A	[123]
Schistosomiasis	190 (180, 200)	N/A	N/A	N/A	[123]
HIV/AIDS	38 (31.6, 44.5)	1.8 (1.3, 2.2)		36.2 (30.2, 42.5)	[124]
Tuberculosis	10.0 (8.99, 11.1)	1.12 (0.98, 1.25)		8.9 (7.85, 9.94)	[125]
Nontyphoidal Salmonellae (NTS)	0.54 (0.41, 0.71)	0.22 (0.16, 0.37)	0.12 (0.07, 0.18)	180.3^2^	[126]

CI, confidence interval. N/A: data not available.

1 Data estimates from sub-Saharan Africa only.

2 Sum of estimates for all age groups ≥14 y.

**Table 5:** Mechanisms of anemia in infectious diseases

Anemia etiology	Infection	Reference
Anemia of inflammation	Chronic infections	[143]
Hemolysis	Malaria, schistosomiasis, HIV, bacterial hemolysins	[147,148, 152]
Blood loss	Hookworm, schistosomiasis, HIV/AIDS	[147,149]
Ineffective RBC production	Malaria, HIV	[148,151, 152]
Pure red cell aplasia	B19 parvovirus-associated	[144]
Nutrient malabsorption	Hookworm, *H. pylori,* gut Microbiome^1^	[149,150, 218]
Hypersplenism	Malaria, HCV, tuberculosis, schistosomiasis	[145,148, 199]
Drug treatment^2^	HCV, HIV, tuberculosis, bacterial infections	[152–154, 196]

HCV, hepatitis C virus; RBC, red blood cell.

1 The gut microbiome facilitates absorption of nutrients such as vitamin B12, thiamin, and folate, but may also contribute to risk and pathogenesis of iron deficiency anemia in populations with low dietary iron intake.

2 Some of the drugs that may cause anemia include antiretroviral therapy (such as zidovudine) and antibiotics (such as cephalosporins).

## Abbreviations used

FPN: ferroportin
G6PD: glucose-6-phosphate dehydrogenase
HAMP: hepcidin antimicrobial peptide
Hb: hemoglobin
HCV: Hepatitis C virus
ID: iron deficiency
RBC: red blood cells
Tb: tuberculosis
