# Supplementary material for: Biology of Anemia: A Public Health Perspective
Source: J Nutr. Author manuscript; Available in PMC 2025 Oct 14. (PMC7618251; doi:10.1016/j.tjnut.2023.07.018)
Supplement: Supplementary Material [file EMS193557-supplement-Supplementary_Material.docx]

JN-2023-0097 - Supplemental_Material

Title: Biology of Anemia: A Public Health Perspective

**Authors**: Gary M. Brittenham^1^, Gemma Moir-Meyer^2,7^, Kelvin Mokaya Abuga^3^, Ananya Datta Mitra^4^, Carla Cerami^5^, Ralph Green^4^, Sant-Rayn Pasricha^2,7,8^, Sarah Atkinson^3,6^

Supplemental Figure Legends:

Supplemental Figure 1. The Approximate Geographical Distribution of the Origins of the β-Thalassemias

Source: Weatherall 2018, page 167 (209). | The specific mutations shown are detailed in the reference.


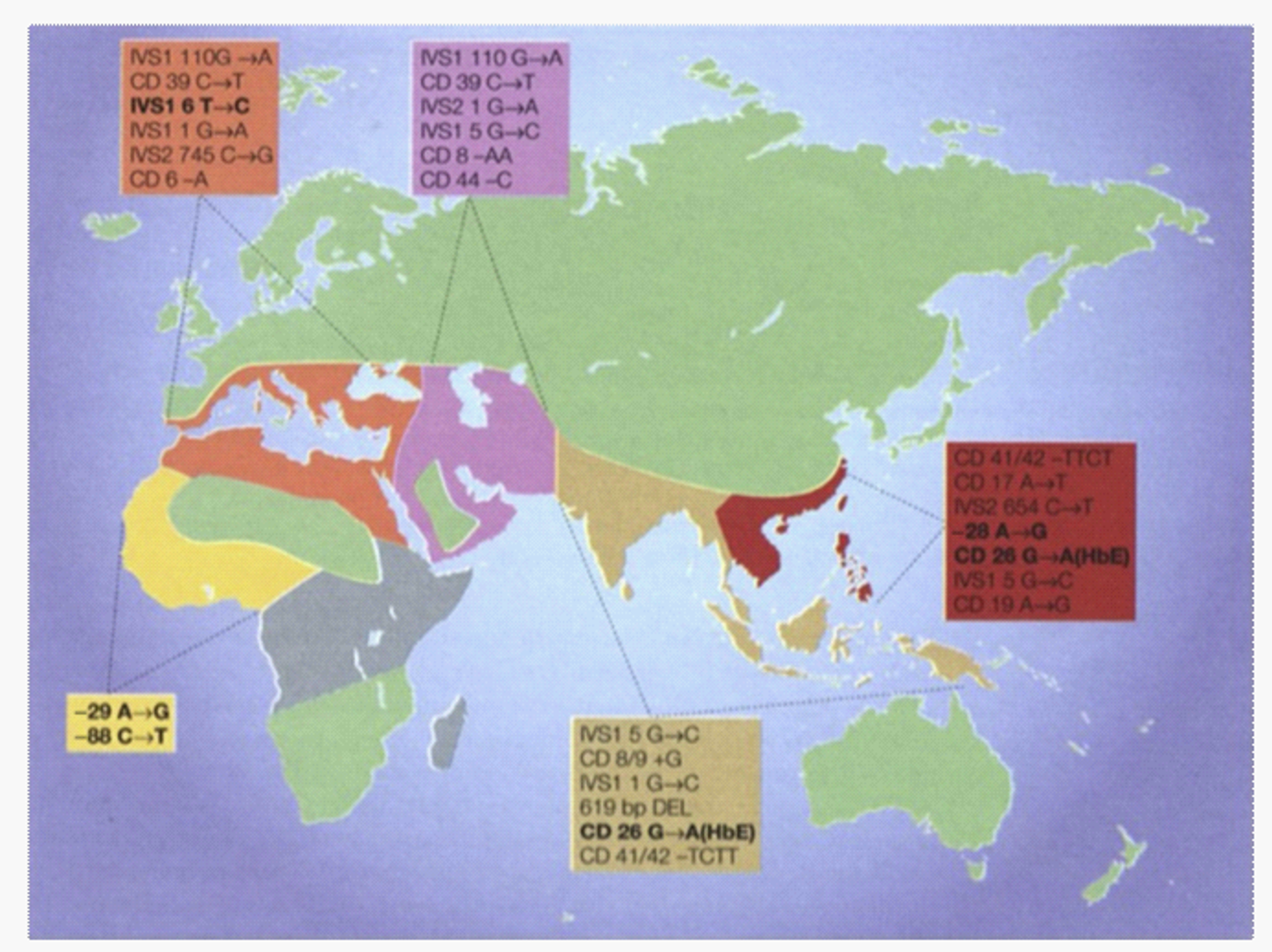


Supplemental Figure 2. The Approximate Geographical Distribution of the Origins of the α Thalassemias

Source: Weatherall 2018, page 166 (209) | The specific mutations shown are detailed in the reference.


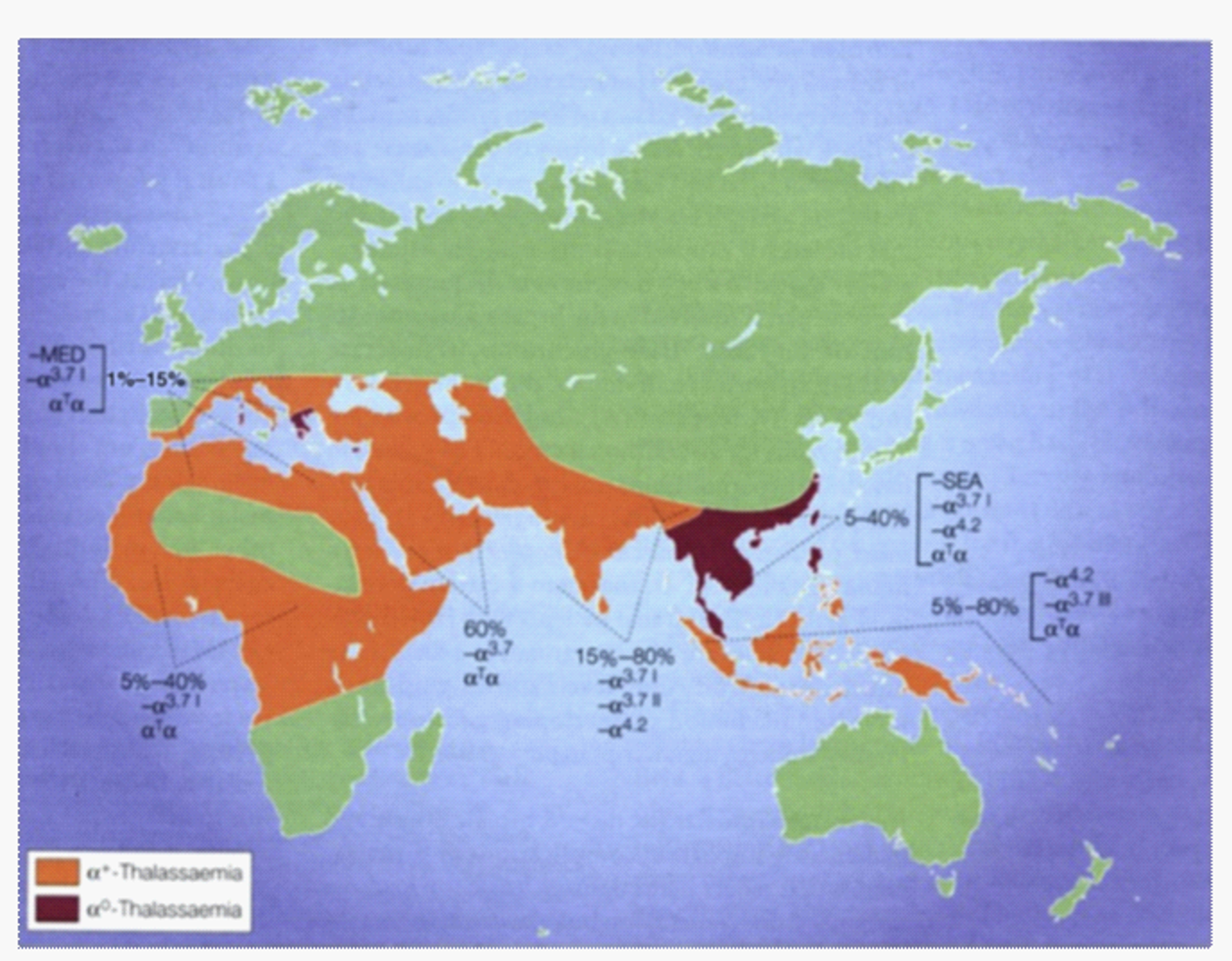


Supplemental Figure 3. The Approximate Geographical Distribution of Hemoglobin S, C, and E

Source: Piel 2016, page 326 (208).


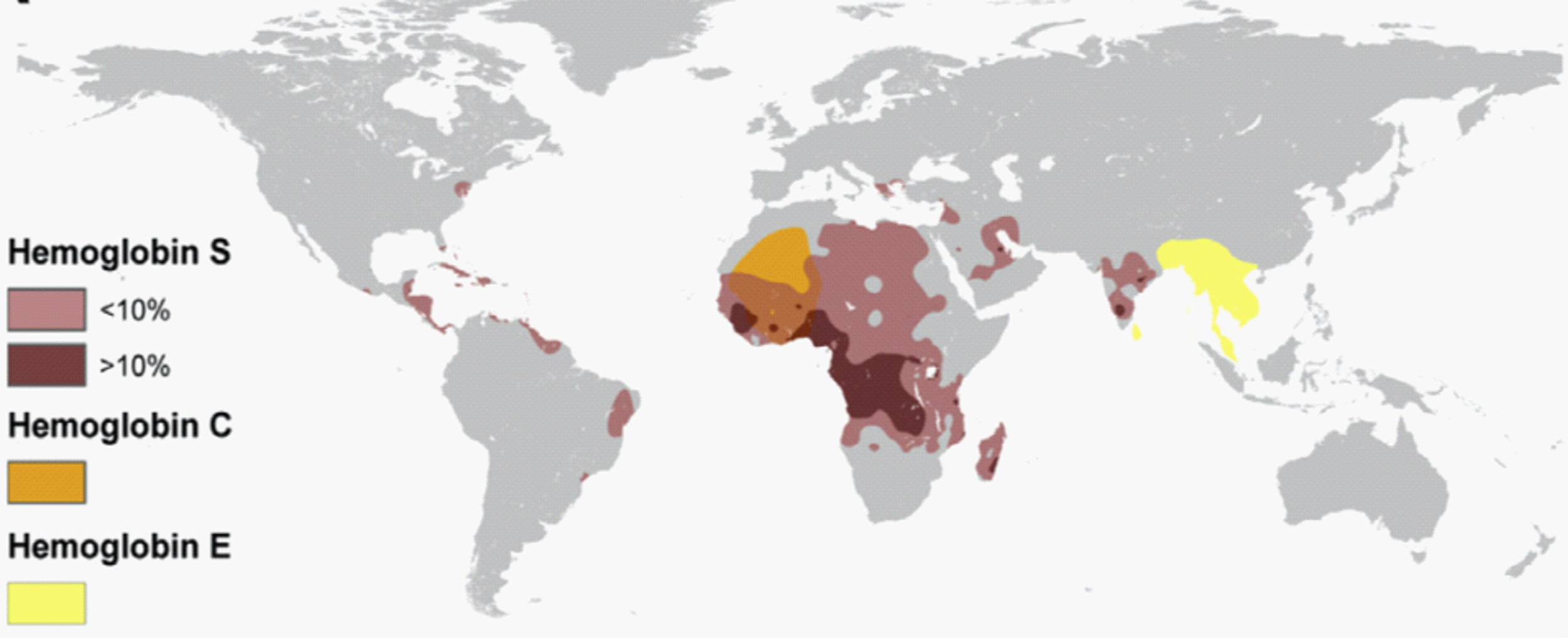


Supplemental Figure 4. The Approximate Geographical Distribution of the Median Predicted Allele Frequency of Glucose-6-phosphatedehydrogenase Deficiency

Source: Howes et al. 2012, page 339 (210).


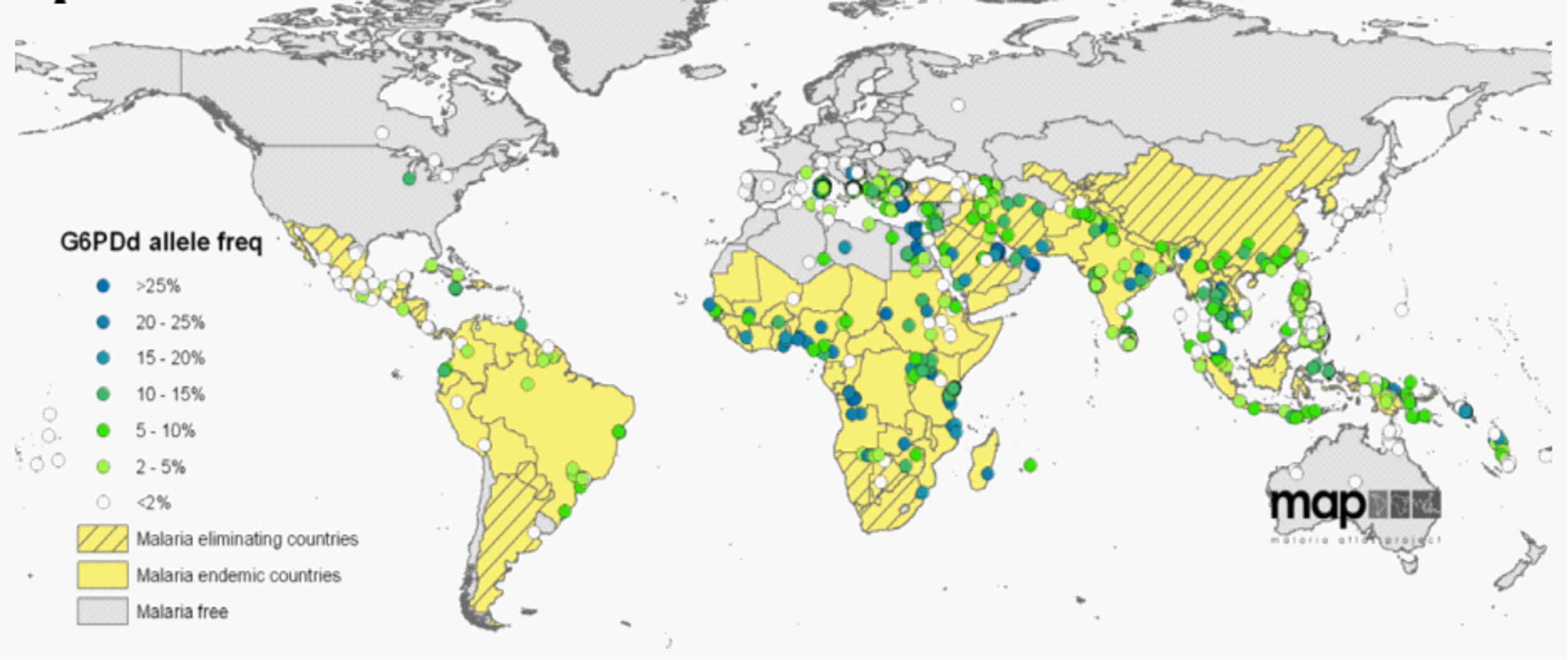


Supplemental Figure 5. Globin Synthesis at the Molecular Level

Hemoglobin synthesis is controlled by two multigene clusters on chromosome 11 (encoding the β­like globins) and on chromosome 16 (encoding the α­like globins). In adult life, the major hemoglobin consists of two different pairs of α-and β-globin, each containing a single heme with an iron atom that reversibly binds oxygen for transport from the lungs to tissues. **(A)** Organization of the β-globin family on chromosome 11 and the α-globin family on chromosome 16. **(B)** The sites of erythropoiesis and the pattern of globin synthesis during development. ψ designates non-expressing pseudogenes. The three exons of the globin genes are shown in pale blue (Taher, Weatherall, and Cappellini 2018, page 156) (212).


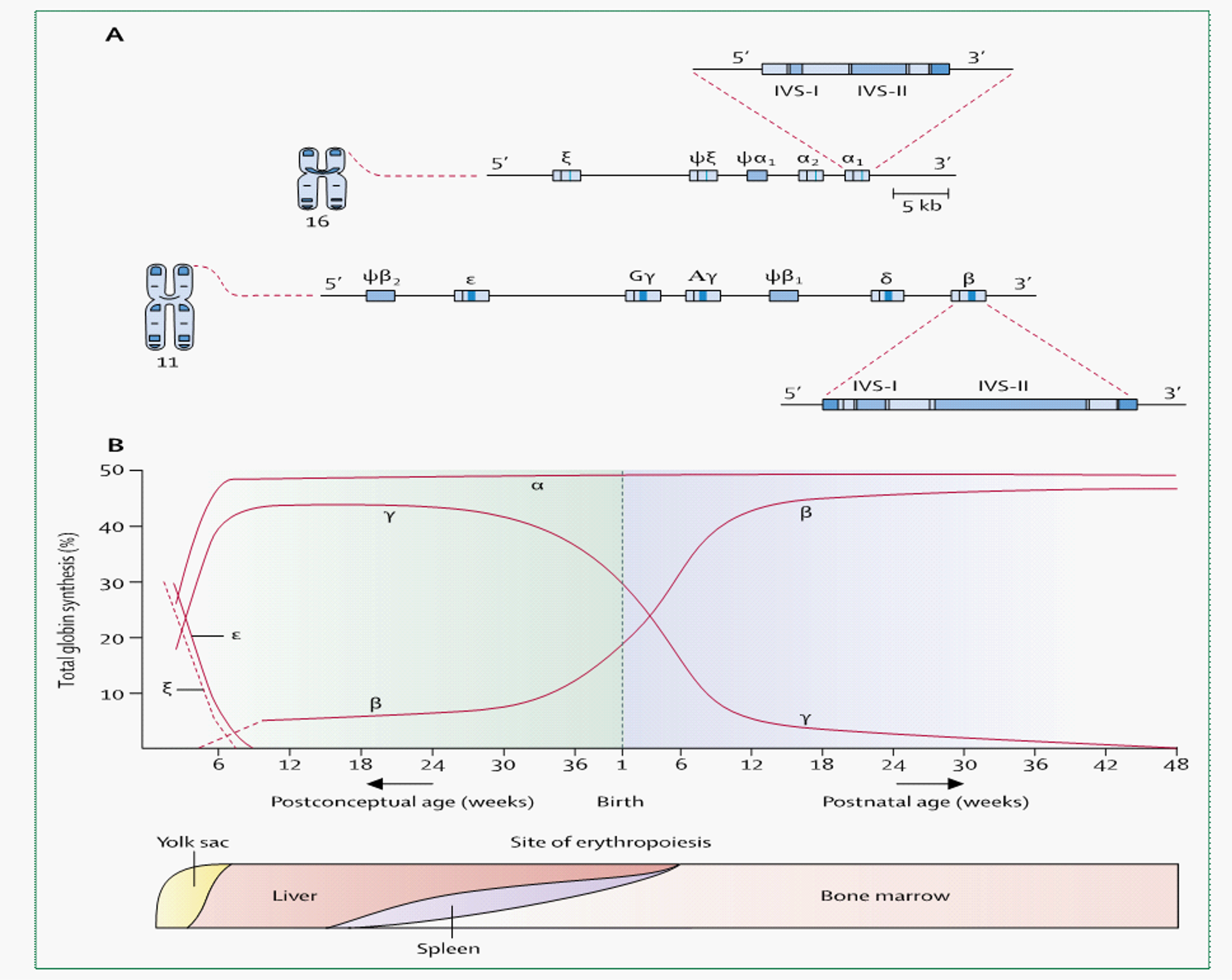


Supplemental Figure 6. Spectrum of Transfusion Requirements in Thalassemia Syndromes

The severity of anemia resulting from thalassemia depends on the type and the pattern of inheritance.

Source: Vichinsky 2016, page 195 (214).


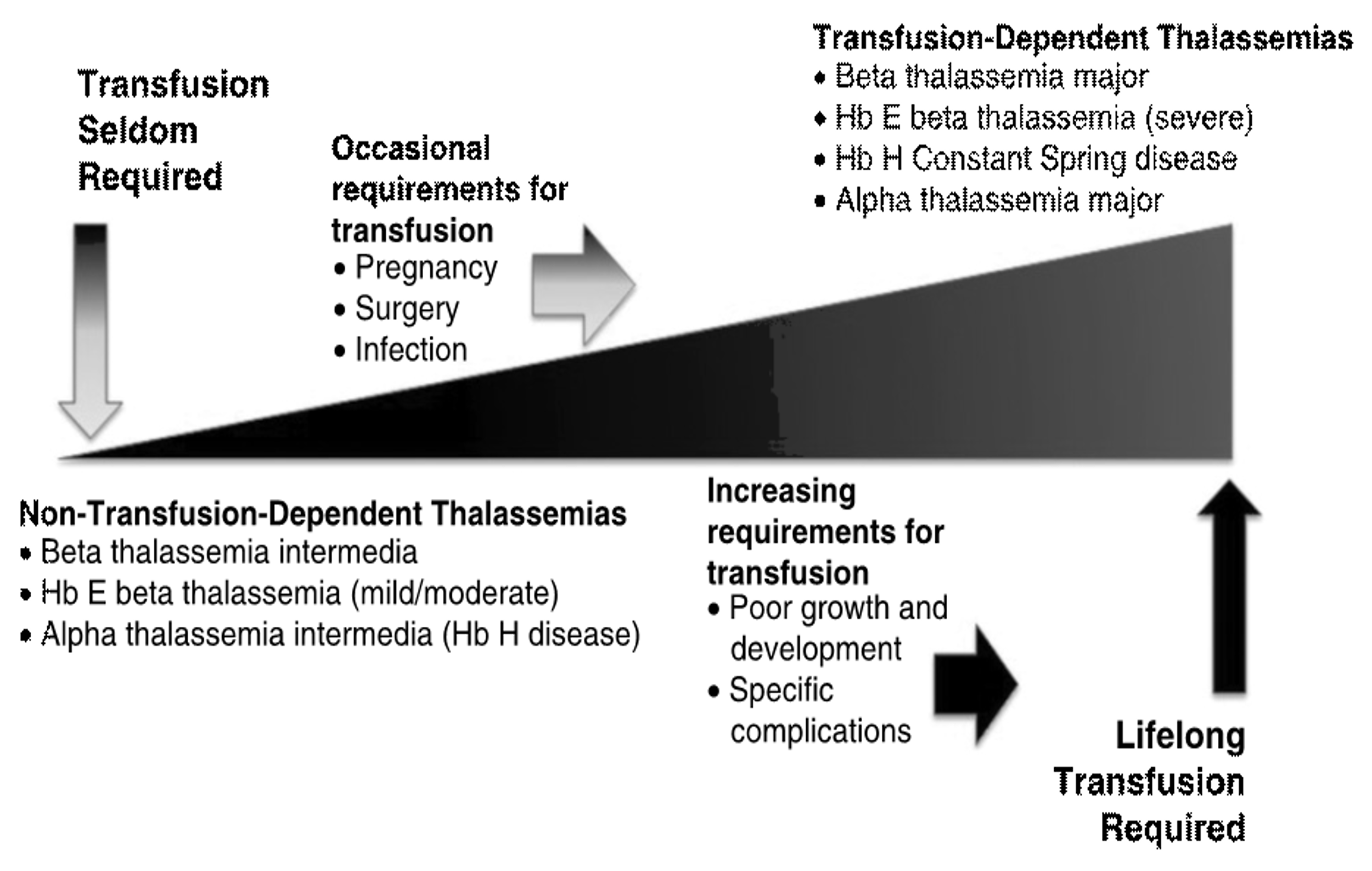


Supplemental Table 1. Interactions between Genetic Red Blood Cell Variants and *Plasmodium* spp. Parasites

| Disorder | Effects |
| --- | --- |
| β-Globin variants | |
| Hb S | > 90% reduction in risk of severe falciparum malaria by HbAS; > 30% reduction in risk of uncomplicated falciparum malaria by HbAS |
| Hb C | 73-86% reduction in risk of severe falciparum malaria by HbCC |
| Hb E | Possible protection from severe falciparum malaria by HbAE and HbEE |
| Hb F | Reduced P. falciparum growth in red cells with high HbF content; reduced cytoadherence of infected red cells with high HbF content |
| β-Thalassemia | Reduced risk of uncomplicated falciparum malaria |
| α-Globin variants | |
| α^+^ Trait | Small reduction in risk of severe falciparum malaria |
| α^0^ Trait | 38% reduction in risk of severe falciparum malaria |
| Surface receptor polymorphisms | |
| ABO blood group | 66% reduction in risk of severe falciparum malaria by blood group o |
| Duffy antigen receptor for chemokines (DARC) | Reduced invasion of DARC-negative, fyfy, red cells by P. vivax in vitro |
| Complement receptor 1 | Reduced risk of severe falciparum malaria with HL alleles |
| Enzymopathies | |
| G6PD deficiency | Reduced risk of severe falciparum malaria in hemizygous males and homozygous females |
| Pyruvate kinase deficiency | Reduced *P. falciparum* invasion of deficient red cells and enhanced clearance of infected red cells in vitro |
| Cytoskeletal abnormalities | |
| Hereditary spherocytosis | Reduced *P. falciparum* growth in vitro |
| Hereditary elliptocytosis | Reduced *P. falciparum* growth in vitro |
| Southeast Asian ovalocytosis | Protection from cerebral falciparum malaria |

Hb AE: heterozygous HbE; Hb AS: sickle Hb; Hb EE: homozygous Hb E; Hb CC: homozygous Hb C; Hb F: fetal Hb; HL: high (H) and low (L) complement receptor 1 expression haplotypes that are codominant and are associated with complement receptor 1 levels on red blood cells. Source: Adapted from Taylor and Fairhurst 2014, page 195 (211).

Supplemental Table 2. Sickle Cell Disease Syndromes and Related Hemoglobinopathies by Genotype

|  | HbA (%) | HbS (%) | HbC (%) | HbF (%) | HbA_2_ (%) | Clinical course | Prevalence (%) |
| --- | --- | --- | --- | --- | --- | --- | --- |
| Normal | 95-98% | 0 | 0 | <1% | <3.5% | - | - |
| Trait conditions | | | | | | | |
| Sickle trait HbAS | 55-65% | 30-40% | 0 | <1% | <3.5% | Benign | 1-8% |
| Haemoglobin C trait | 55-65% | 0 | 30-40% | <1% | <3.5% | Benign | 1-3% |
| Β-thalassaemia trait | 90-95% | 0 | 0 | 1-3% | >3.5% | Benign | 1-2% |
| Disease conditions | | | | | | | |
| Sickle cell anaemia | 0 | 80-95% | 0 | 5-15% | <3.5% | Severe | 50-60% |
| Sickle-C disease | 0 | 50-55% | 40-45% | <3% | <3.5% | Moderate | 25-30% |
| S/β^0^ thalassaemiat | 0 | 80-90% | 0 | 5-15% | >3.5% | Severe | 1-3% |
| S/β^+^ thalassaemia | 10-25% | 70-80% | 0 | <3% | >3.5% | Mild | 5-10% |
| S/ Other (Hb variant) | 0 | 50-60% | 0 | Variable | <3.5% | Variable | 1-2% |

Source: Ware et al. 2017, page 2 (217).
